# Anti-Fouling Double-Skinned Forward Osmosis Membrane with Zwitterionic Brush for Oily Wastewater Treatment

**DOI:** 10.1038/s41598-017-07369-4

**Published:** 2017-07-31

**Authors:** Chi Siang Ong, Bader Al-anzi, Woei Jye Lau, Pei Sean Goh, Gwo Sung Lai, Ahmad Fauzi Ismail, Yue Seong Ong

**Affiliations:** 1 Department of Environment Technology and Management, College of Life Sciences, Kuwait University, Kuwait; 2 Advanced Membrane Technology Research Centre (AMTEC), Universiti Teknologi Malaysia, 81310 Skudai, Johor Malaysia; 3 Faculty of Engineering and the Built Environment, SEGi University, 47810 Petaling Jaya, Selangor Malaysia

## Abstract

Despite its attractive features for energy saving separation, the performance of forward osmosis (FO) has been restricted by internal concentration polarization and fast fouling propensity that occur in the membrane sublayer. These problems have significantly affected the membrane performance when treating highly contaminated oily wastewater. In this study, a novel double-skinned FO membrane with excellent anti-fouling properties has been developed for emulsified oil-water treatment. The double-skinned FO membrane comprises a fully porous sublayer sandwiched between a highly dense polyamide (PA) layer for salt rejection and a fairly loose dense bottom zwitterionic layer for emulsified oil particle removal. The top dense PA layer was synthesized via interfacial polymerization meanwhile the bottom layer was made up of a zwitterionic polyelectrolyte brush - (poly(3-(N-2-methacryloxyethyl-N,N-dimethyl) ammonatopropanesultone), abbreviated as PMAPS layer. The resultant double-skinned membrane exhibited a high water flux of 13.7 ± 0.3 L/m^2^.h and reverse salt transport of 1.6 ± 0.2 g/m^2^.h under FO mode using 2 M NaCl as the draw solution and emulsified oily solution as the feed. The double-skinned membrane outperforms the single-skinned membrane with much lower fouling propensity for emulsified oil-water separation.

## Introduction

Oily wastewater is a mixture of oil, solids and water produced from petroleum and petrochemical industries or other oil-related activities. With rapid industrial development, there is an increase in the amount of oil used which might produce a large quantity of oily wastewater and cause potential risk to water environment due to the existence of many hazardous hydrocarbon mixture, chemical components and heavy metals^1, 2^. The effluent discharged from those oil productions might pose a potential risk to our nature water bodies system hence an urgent action is needed to tackle the problem. In view of this, different strategies include gravity settling^3^, coagulation^4^, air flotation^5^, fibrous/packed bed coalescence^6^ and membrane-based technologies^7–10^ have been developed to meet the stringent oil discharge standard established in different countries. Of the treatment methods, membrane-based technology has received a lot of attention from both academia and industry owing to its unique characteristics such as small footprint area, low energy consumption, high separation efficiency and a relatively simple process from an operational viewpoint^11, 12^.

Compared to the pressure-driven reverse osmosis (RO) and nanofiltration (NF) membrane processses, forward osmosis (FO) membrane process that utilizes an osmotic pressure gradient to extract water across a semi-permeable membrane from a feed solution (FS) of lower osmotic pressure to a draw solution of higher osmotic pressure requires significantly lower energy consumption during operation. This process is hypothesized to show lower fouling potential in the case where the active rejection layer of membrane is arranged to face foulant-containing feed solution, i.e., active layer-facing-feed–solution (AL-FS) orientation^13, 14^. However, the major drawback of AL-FS orientation is the dilutive internal concentration polarization (ICP) that results in a reduced concentration of draw solutes inside the porous support^15, 16^. When the active layer faces the draw solution (AL-DS orientation), the feed solutes rejected by the selective layer accumulate in the membrane support, causing the concentrative ICP. In comparison, the AL-FS orientation has better fouling-resistance but with lower initial water flux^13^. Therefore, conventional asymmetric single-skinned FO membranes face a dilemma of either experiencing more severe dilutive ICP in AL-FS or having much higher fouling propensity in AL-DS^17^.

This dilemma can be resolved by designing a double-skinned membrane structure (i.e., a porous support sandwiched between two rejection skins), with a dense rejection skin facing the draw solution to prevent solute reverse diffusion and with a second rejection skin on the feed solution side for fouling prevention^18–27^. Zhang *et al*.^28^ studied the antifouling properties using a double-skinned cellulose acetate (CA) FO membrane and showed higher restoration of the water flux by simple membrane cleaning process. Qi *et al*.^22^ deposited additional skins using layer-by-layer technique and demonstrated superior anti-fouling properties. Duong and Chung^24^ also reported that oil-emulsion separation could be achieved by FO process using a double-skinned thin film composite (TFC) membrane with a superhydrophilic Nexar^®^ copolymer coating layer. It was reported that pure water could be recycled from the extremely concentrated oily wastewater (200,000 ppm) at a high water flux of 10.9 L/m^2^.hand high-quality pure water (with oil rejection >99.9%) under AL-DS mode using 0.5 M NaCl as the draw solution. However, the authors did not mention about the water flux under FO mode. The most recent studies by Tang *et al*.^21^ and Zhou *et al*.^18^ however reported that double-skinned TFC membranes have minimum effect on eliminating the ICP occurred in the membrane support layer, and lower water flux was obtained compared to single-skinned TFC membrane. The water flux of double-skinned membranes was also reported to decline greater using a more concentrated salt solution during FO process. Thus, the advantages of double-skinned membrane over single-skinned membrane still remain debatable, which could be affected by the anti-fouling material deposited on the porous substrate and targeting feed water solution.

Zwitterionic polymers have excellent antifouling ability and have been postulated as a new generation of antifouling materials^29–32^. The low fouling behaviour of zwitterionic materials is attributed to their ability to bind a significant number of water molecules through both electrostatic and hydrogen bonding interactions, and the water molecules bond with the zwitterionic materials will form the strong hydration layer and effectively prevent the oil adhesion on membrane surface^33, 34^. Recently, surface grafting zwitterionic monomers has become an attractive way to modify ultrafiltration (UF) membranes.

For instance, Zhu *et al*.^33^ reported a novel polyvinylidene fluoride (PVDF) membrane grafted with zwitterionic polyelectrolyte brush - (poly(3-(*N*-2-methacryloxyethyl-*N*,*N*-dimethyl) ammonatopropanesultone) (PMAPS) via surface-initiated atom transfer radical polymerization process. The modified membrane showed extremely high water flux and hydrophilicity upon 12-h polymerization. They attributed the superior hydrophilicity and flux performance to the higher surface energy and hydrated behavior of zwitterionic polyelectrolyte polymers in water. Similar results were also reported by other researchers in which zwitterion-coated membranes exhibited improved anti-fouling performance and greater flux recovery rate^35–37^.

The objective of the current study was to fabricate double-skinned polyamide (PA) TFC membranes by incorporating in-house synthesized zwitterionic polymer at the bottom layer. The FO performances and fouling behaviors of both single and double-skinned TFC membranes were investigated. The effects of zwitterionic polymers on the antifouling properties of TFC membranes in oily wastewater treatment process were also investigated in order to provide a useful platform to design FO membranes for highly foulant contaminated feeds. To the best of our knowledge, this is the first attempt to fabricate double-skinned zwitterionic TFC membranes for oily waste water treatment.

## Experimental

### Materials

Polyethersulfone (PES) in pellet form (Solvay Specialty Polymers), N-Methyl-2-pyrrolidone (purity >99.5%, RCl-Labscan) and polyvinylpyrrolidone (PVP K30, Sigma-Aldrich) as additive were used for fabrication of membrane substrate. 1,3-phenylenediamine (MPD) and trimesoyl chloride (TMC) purchased from Sigma Aldrich were used to form a PA layer on top of the PES substrate. To synthesize zwitterion properties polymer, 2-(dimethylamino)ethyl methacrylate (DMAEMA, 95%), 2-bromoisobutyrate bromide, diisobutylaluminium hydride (1.0 M in toluene), ethyl 2-bromoisobutyrate (EBiB, 98%), 1,3-propanesultone (99%), dipyridyl (Bpy, AR), 2-bromoisobutyryl bromide (98%), copper(I) bromide (CuBr, 99%) and 1-hexyl-3-methylimidazolium chloride (HMImCl) were purchased from Sigma Aldrich. Diethyl ether was purchased from RCl-Labscan. Sodium chloride (NaCl, Merck) was used as test solute for membrane flux and rejection determination. The feed solution containing the respective test solute was prepared by dissolving the solute in deionized (DI) water.

## Membrane Preparation

A porous PES flat sheet membrane support was fabricated using the phase inversion method described in literature^38^. The dope solution used for support fabrication contained 18 wt.% PES, 1 wt.% PVP and 81 wt.% NMP. The detailed procedures for the casting conditions and post-treatment of the PES membrane substrate can be found in the SI.

**Figure 1:**
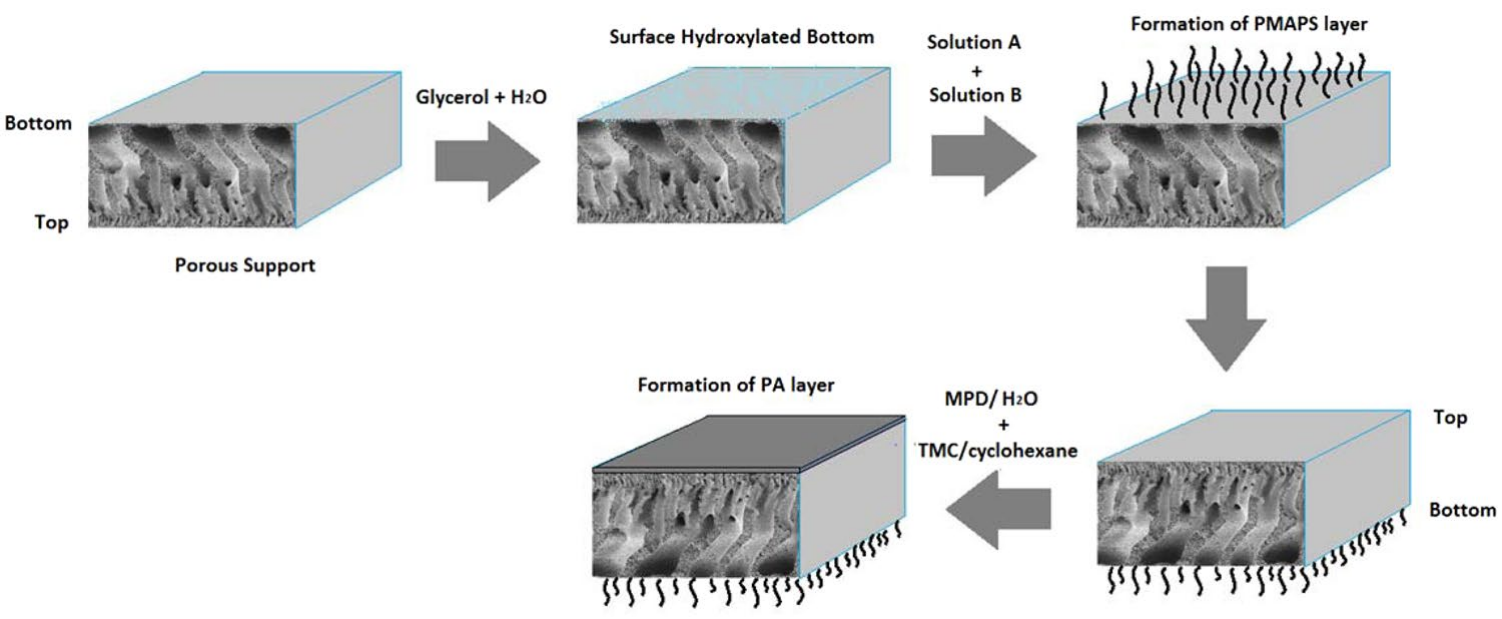
The fabrication procedure of double-skinned TFC membrane.

To fabricate the single-skinned TFC FO membrane, a PA layer was deposited onto the top of the PES support by the interfacial polymerization process between MPD and TMC monomers, as described in the SI. Meanwhile for the double-skinned FO membrane, the zwitterionic copolymer layer was first formed onto the bottom of the support following by the formation of the PA layer on the top of the support as illustrated in Fig. 1. The detailed synthesis process of zwitterionic co-polymer is disclosed in the SI.

## Membrane Characterization

Cross sectional and surface images of the composite membranes together with elemental analysis were obtained using a Hitachi SU8000 field emission scanning electron microscope (FESEM). Prior to the analysis, the double-skinned TFC membrane was immersed into liquid nitrogen for a few minutes followed by freeze fracturing to obtain a perfect cut structure. The fiber was then placed onto a carbon-tape aluminum holder and coated with platinum under vacuum. The surface roughness of the membrane was studied by using non-contact atomic force microscopy (AFM) mode (Park System XE-100) in order to characterize the surface roughness of top and bottom layer of double skinned membranes. A small piece of double skinned membrane was cut and adhered on a 1 cm^2^ square paper card using double-sided adhesive tape. The membrane surface was scanned in the size of 5 μm × 5 μm. Fourier transform infrared spectroscopy (FTIR) spectra of the membranes were recorded in the mid IR region between 700 cm^−1^ and 4000 cm^−1^ with an average of 16 scans and at a resolution of 4 cm^−1^. by using Perkin Elmer Spectrum One FTIR Spectrophotometer. The contact angle of the membrane was measured by using the contact angle goniometer (Attension/KSV Theta, Japan). Approximate 3 µL of DI water was dropped on the bottom surface of both single and double skinned membrane samples. The contact angle was measured from the water-membrane interface. At least 10 locations were arbitrarily chosen in order to yield an average value.

## Performance Evaluation

### Pure Water Permeability, Salt Permeability, and Salt Rejection Tests

All the tests were conducted using a commercial stirred dead-end permeation cell (HP4750, Sterlitech Corp.). The membrane was placed in the permeation cell with a minimum 80% of water level. All membranes were compacted for 30 min at 10 bar to obtain a steady state flux prior to testing. After that, the water permeate was collected at a fixed interval of 20 min to obtain a measureable amount of water volume at 9 bar. This was repeated 3 times to obtain an average reading. The pure water permeability coefficient, A (Lm^−2^h^−1^bar^−1^) was calculated according to Equation (1).

1 $$A=\frac{V\times 60}{{A}_{eff}\times t\times 1000\times \Delta P}$$

where *V* is the volume (mL), *t* is time (min), *A*_*eff*_ is the effective membrane area (14.6 cm^2^), and *ΔP* is the applied pressure difference.

In salt flux and salt rejection test, 2000 ppm NaCl solution was used. DI water was used to compress the membrane before the test was carried out. The system was allowed to run for 15 min prior to the experiment in order to ensure the permeate water quality and flux stability. This was repeated 3 times to obtain an average value. A conductivity meter (HC3010, Trans Instruments) was used to measure the salt conductivity. The conductivity of the permeate (*C*_*p*_) and feed (*C*_*f*_) were measured 3 times to obtain an average value. The salt permeability coefficient, *B* (Lm^−2^h^−1^bar^−1^), and salt rejection, *R* (%) were calculated according to Equations (2) and (3), respectively,

2 $$B=\frac{V\times 60}{{A}_{eff}\times t\times 1000\times \Delta {P}}$$

3 $$R=(1-\frac{{C}_{p}}{{C}_{f}})\times 100 \% $$

### Oil Flux, Oil Rejection, and Anti-Fouling Performance Test

The DI and salt water used in the previous study was replaced with 10,000 ppm commercial oil (Red Eagle, Malaysia) in water. The preparation of oil emulsion feed solution is described in the SI. The system was allowed to run for 15 min before any value was recorded. The permeate was collected at a fixed interval to obtain a measureable amount of water volume over a period of 30 min using RO testing. The oil concentrations in permeate and feed were determined using a UV-vis spectrophotometer (DR2800, Hach) with absorbance measured at 294 nm which the maximum absorption occurs. The relation between absorbance and oil concentration is found to be linear as shown in Figure S1. The same relation has been used for measuring unknown oil concentration in each permeate.

The oil flux, *J*_*o*_ (Lm^−2^h^−1^) was calculated using the average of the first 3 intervals according to Equation (4),

4 $${J}_{o}=\frac{V\times 60}{{A}_{eff}\times t\times 1000}$$

where *V* is the volume (mL), *t* is time (min), and *A*_*eff*_ is the effective membrane area (14.6 cm^2^).

The oil rejection, *R*_*o*_ (%) was calculated according to Equation (5).

5 $${R}_{o}=(1-\frac{{C}_{p}}{{C}_{f}})\times 100 \% $$

where *C*_*f*_ (ppm) and *C*_*p*_ (ppm) are the concentration of feed and permeate, respectively.

After the oil filtration test, the membrane was removed from the system and cleaned by DI water for 30 min. The anti-fouling testing was conducted in RO mode for both DI water and oil water solution over a period of 480 min to observe the flux change along the experiment.

### Forward Osmosis Test

**Figure 2:**
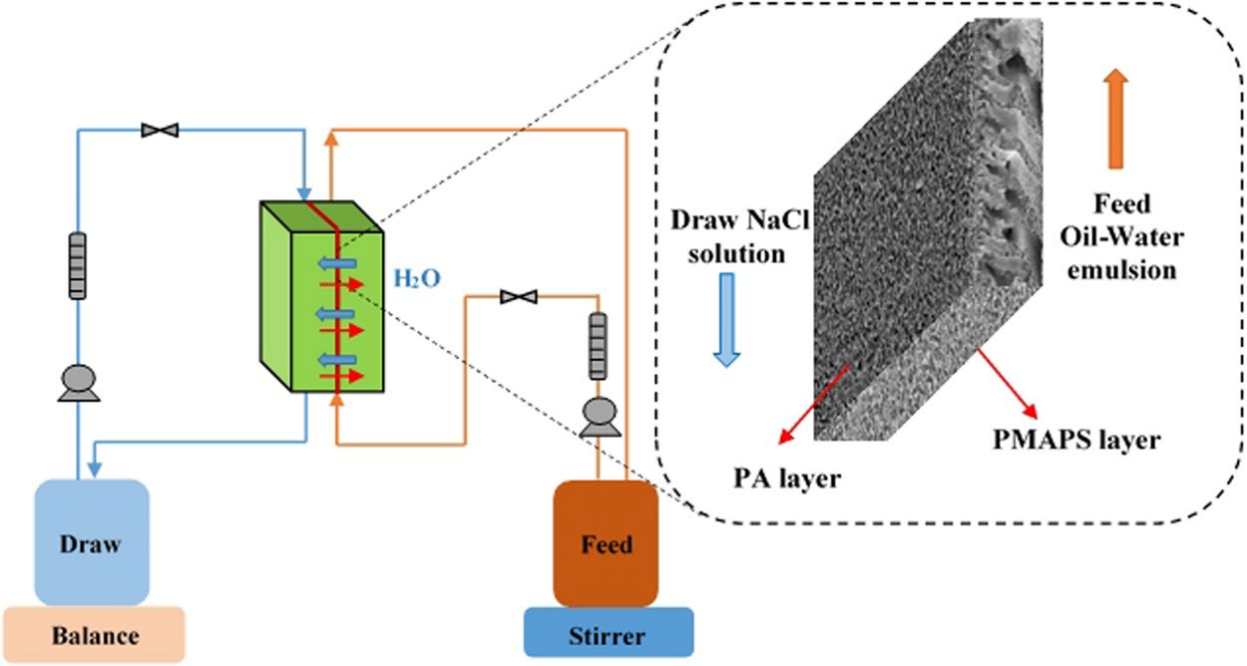
Schematic diagram of the lab-scale FO set-up for testing of the double-skinned membrane.

Figure 2 shows the schematic diagram of the lab-scale FO set-up for testing of double-skinned membrane. The designed lab-scale FO system has a total effective membrane area of 42 cm^2^. Two variable micro gear pumps (Longer Pump, China) were used to circulate the feed and draw solutions. The pressure and temperature were maintained at atmospheric pressure and ambient temperature, respectively. Both flow velocities of feed solution and draw solution were fixed at 1.1 L/min. The water flux of membrane was evaluated by measuring the difference in the volume before and after the experiment. A conductivity meter was used to measure the solution conductivity of the feed solution. An UV-vis spectrometer was used to measure the oil concentration of the draw solution.

**Figure 3:**
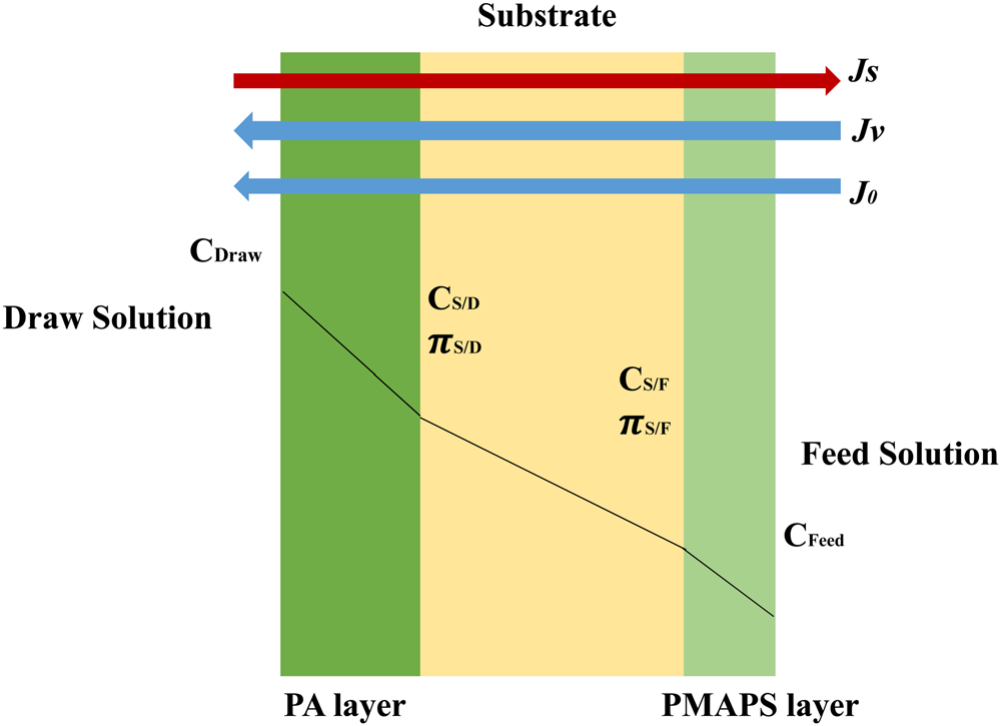
Concentration profile of double-skinned membrane.

All the membranes were tested under AL-DS mode, where the PA active layer faces to draw solution and PMAPS layer faces to feed oil-water emulsion. Figure 3 shows the concentration profile of the double-skinned membrane. The system was allowed to run for 30 min to stabilize the flows prior to the testing. The experiment was conducted for 1 h. An oil concentration of 10,000 ppm commercial oil was used as feed solution while 2 M NaCl was used as draw solution. The FO water flux, *Jv* (L/m^2^.h, abbreviated as LMH) was calculated according to Equation (6) where *ΔV* is the volume change of feed solution (mL), *A*_*eff*_ is the effective membrane area (cm^2^), and *Δt* is the measuring time interval (h).

6 $${J}_{v}=\frac{{\rm{\Delta }}V}{{A}_{eff}\times {\rm{\Delta }}t\times 1000}$$

The reverse solute flux, *Js* (g/m^2^.h, abbreviated as gMH) was calculated using Equation (7) based on the change in salt concentration in the feed solution where *A*_*eff*_ (~58.7 cm^2^) is the effective membrane area (cm^2^), *Δt* is the time interval (h), *ΔC*_*f*_ (g/L) and *ΔV* (mL) are the change in salt concentration and volume of feed solution measured at the beginning and the end of the time interval, respectively. The conductivity of the feed solution was measured using a conductivity meter and a calibration graph was used to convert the respective values into concentration.

7 $${J}_{s}=\frac{{\rm{\Delta }}{C}_{f}\times {\rm{\Delta }}V}{{A}_{eff}\times {\rm{\Delta }}t\times 1000}$$

The oil flux, *J*_*o,FO*_ (gMH) was calculated using Equation (8) based on the oil concentration in the draw solution where *A*_*eff*_ is the effective membrane area (cm^2^), *Δt* is the time interval (h), *ΔC*_*d*_ (g/L) and *ΔV* (mL) are the change in oil concentration and volume of draw solution measured at the beginning and the end of the time interval, respectively. The absorbance of the draw solution was measured using an UV-vis spectrometer and a calibration graph was used to convert the respective values into concentration.

8 $${J}_{o,FO}=\frac{{\rm{\Delta }}{C}_{d}\times {\rm{\Delta }}V}{{A}_{eff}\times {\rm{\Delta }}t\times 1000}$$

## Results and Discussion

**Figure 4:**
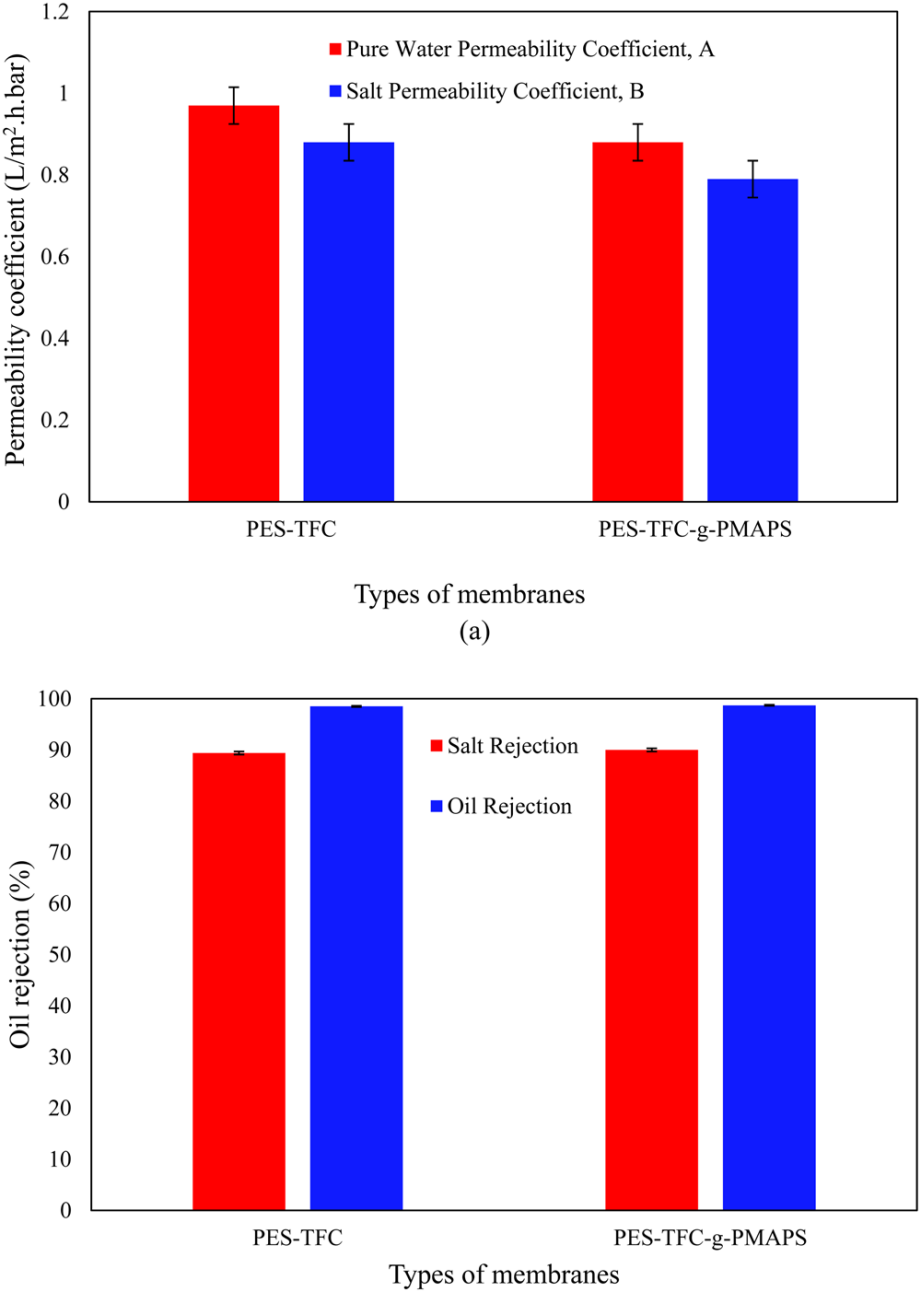
Transport properties of the single-skinned and double-skinned TFC membranes, (**a**) pure water permeability coefficient and salt permeability coefficient and (**b**) salt rejection (Red) and oil rejection (Blue) for DI water and 10,000 ppm oil water solution tested under RO mode at 9 bar.

Figure 4(a) and (b) compare the single- and double-skinned TFC membranes with respect to solvent/solute permeability and rejection. From the figure, the PWP of the single-skinned membrane (0.97 LMH) is slightly higher compared to the double-skinned TFC membrane (0.88 LMH). The performance of the resultant single- and double-skinned TFC membrane was further compared with other membranes reported in the literature and the results are summarized in Table S1. As can be seen, the water fluxes shown in this work are obviously higher compared with the finding of Zhou & Lee (0.59 LMH) where double-skinned CA membrane was used^18^. With respect to the hydrophilicity of both single and double-skinned membranes, it was found that bottom surface of double-skinned membrane has lower contact angle compared to that of single-skinned membrane, recording 64° and 74°, respectively. This indicates that the presence of PMAPS at the bottom surface of PES substrate imparts significant change on the membrane bottom surface hydrophilicity. With respect to rejection, both single- and double-skinned membranes were able to achieve about 90% NaCl removal. The coating of PMAPS on the bottom surface has no role in rejecting ions as no dense layer is established. This is consistent with one of the relevant works that TFC layer controls the salt selectivity of the double-skinned membrane^24^, while the zwitterionic co-polymer serves as an antifouling layer.

Table 1 compares the performance between single- and double-skinned TFC membranes. When using 10,000 ppm emulsified oil-water solution as the feed under AL-DS mode, the water flux of the PES-TFC-g-PMAPS is lower in comparison to the PES-TFC owing to the increased transport resistance created by PMAPS layer. It is worth to mention that a high quality water with purity >99.9% can be drawn from an oily solution using the PES-TFC-g-PMAPS membrane at a reasonably good water flux of 13.7 ± 0.3 LMH using 2 M NaCl as the draw solution. The excellent oil rejection of both membranes can be indicated by the minimum oil flux obtained as shown in Table 1. The PES-TFC-g-PMAPS membrane displayed lower *J*_*v*_ and *J*_*s*_ compared to the PES-TFC membrane due to the presence of PMAPS layer that increases mass transport resistance.

**Table 1:** Performance of the single-skinned (PES-TFC) and double-skinned membrane (PES-TFC-g-PMAPS).

^a^Membrane Designation	Water Flux, *J*_*v*_ (LMH)	Reverse Solute Flux, *J*_*s*_ (LMH)	Oil Flux, J_0_ (gMH)
PES-TFC	14.6 ± 0.2	4.60 ± 0.1	<0.05
PES-TFC-g-PMAPS	13.7 ± 0.3	1.60 ± 0.2	<0.03

Both membranes were evaluated at AL-DS mode; Draw solution: 2 M NaCl; Feed solution: 10,000 ppm of oil water solution.

**Figure 5:**
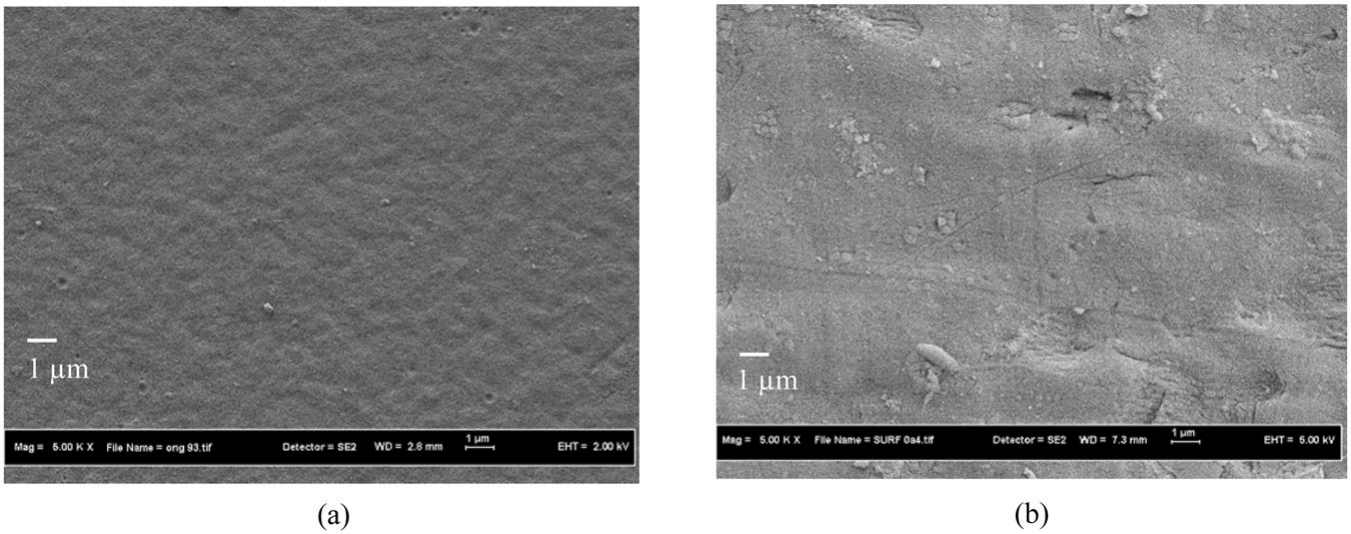
FESEM images of PES substrate, (**a**) top surface and (**b**) bottom surface.

**Figure 6:**
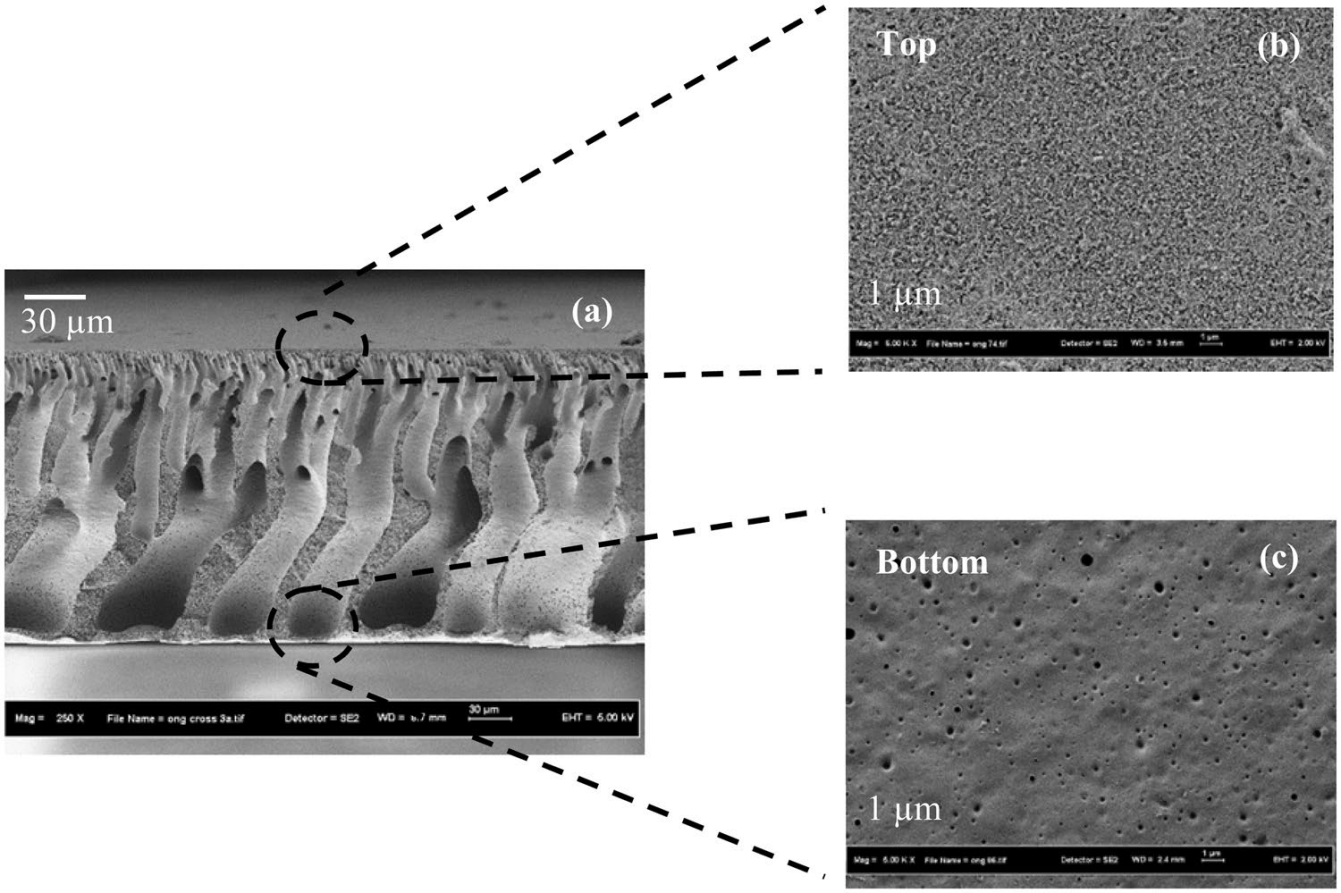
FESEM images of double-skinned TFC membrane, (**a**) cross-sectional morphology, (**b**) top PA selective and (**c**) PMAPS-grafted bottom surface.

**Figure 7:**
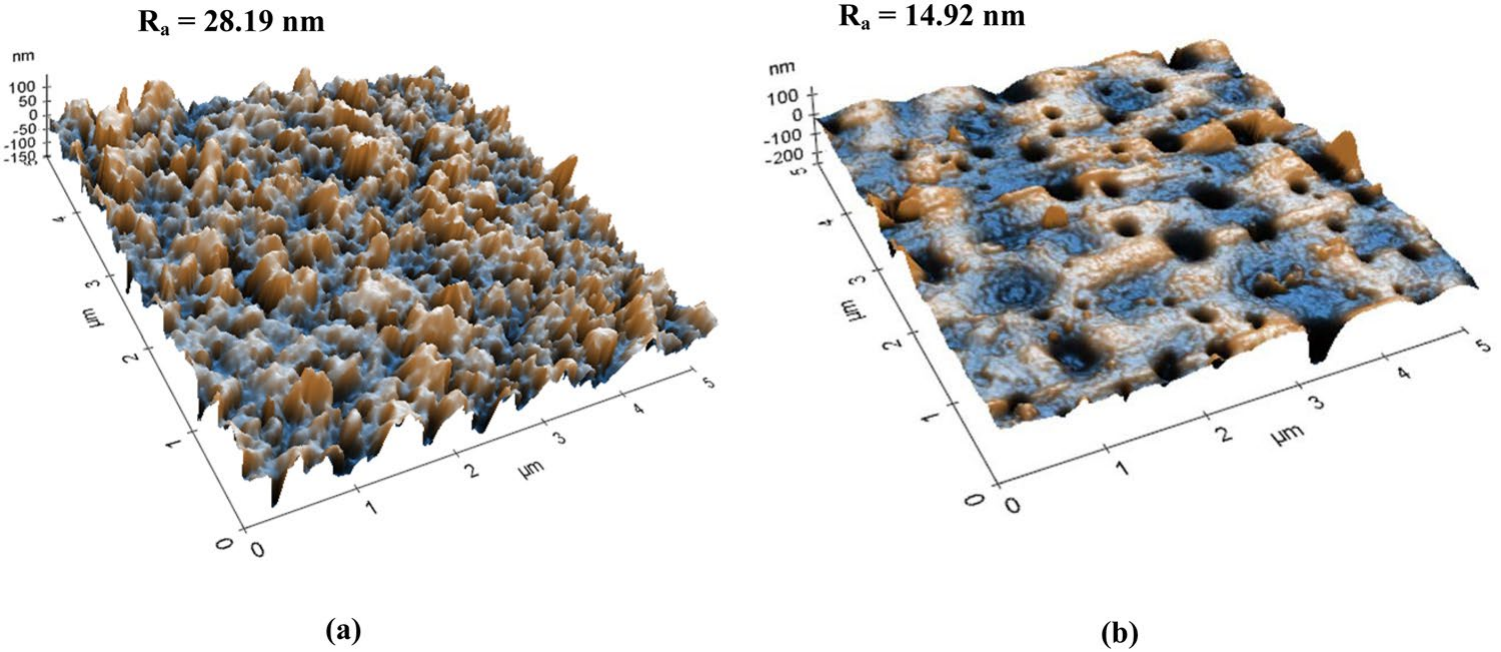
AFM images of (**a**) PA layer and (**b**) PMAPS layer of double-skinned membrane.

Figures 5, 6 and 7 show the FESEM and AFM images of the newly fabricated double-skinned TFC membrane. From the figure, the double-skinned membrane has a highly porous middle layer consisting of long finger-like structure. The formation of such microporous structure is likely to decrease water transport resistance and reduce ICP^39^. A thin layer with rough ridge-valley morphology formed on the top of the support is due to the formation of a PA layer via interfacial polymerization onto the PES substrate. The detection of holes on the bottom surface of PES-TFC-g-PMAPS indicated that PMAPS grafting using 2-bromoisobutyrate bromide as an initiator^33^ tended to increase bottom surface porosity by creating small holes. From the AFM images, it was found that the average surface roughness of bottom PMAPS layer is relatively smaller compared to the top PA layer. The bottom PMAPS layer of double-skinned TFC membrane displayed much lower average surface roughness value of 14.92 nm in comparison with 28.19 nm reported for bottom surface of single-skinned TFC membrane. Previous studies have correlated the changes in membrane anti-fouling properties with the membrane top surface roughness in which the lower the surface roughness value, the better the anti-fouling properties and the greater the flux recovery rate^40, 41^. In contrast, larger membrane surface roughness is also possible to enhance attachment of foulants onto the membrane surface due to larger surface area and greater contact opportunities for particles with the membrane surface^40^. Hence, it is believed that the remarkable decline in surface roughness of double-skinned TFC membrane is one of the possible factors influencing the membrane surface anti-fouling properties due to Cassie-Wenzel effect^41, 42^.

**Figure 8:**
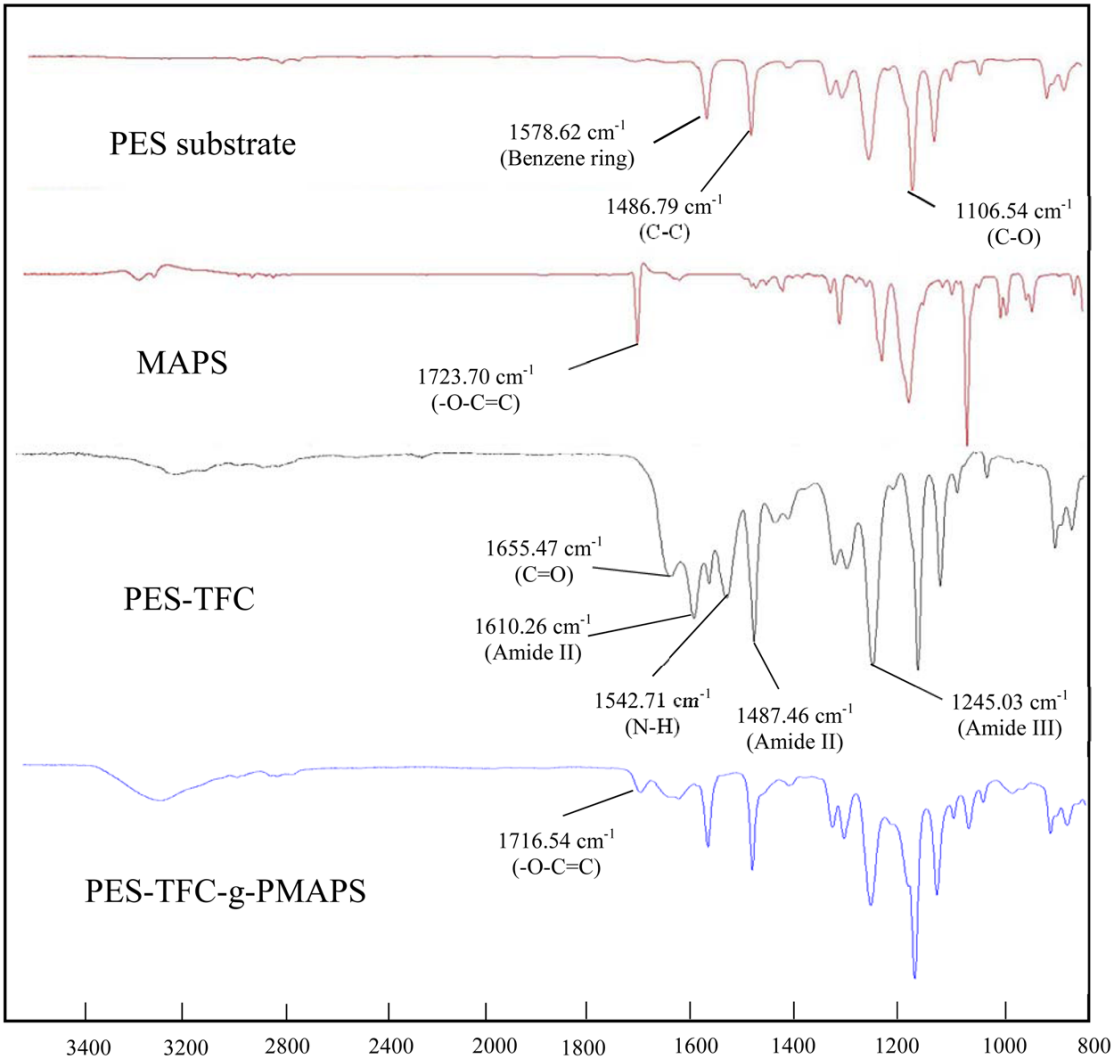
FTIR spectra of membranes.

The successful formation of PA and PMAPS layers onto the PES support were further confirmed by FTIR and the results are shown in Figure 8. The PA main characteristic bands of (amide I) C = O stretching vibrations and (amide-II) N-H band of amide group (-CONH-) are appeared at 1655.47 cm^−1^ and 1542.71 cm^−1^, respectively. Other characteristic bands of PA are found at 1610.26 and 1487.46 cm^−1^ (aromatic ring stretching), and 1245.03 cm^−1^ (amide III)^43^. Separately, the characteristic band of -O-C = C group that belongs to the zwitterion polymer is present at 1716.54~1723.70 cm^−1^ ^33^. This confirms the formation of PMAPS copolymer film onto the PES substrate.

### Prolonged studies of PES-TFC and PES-TFC-g-PMAPS membranes

**Figure 9:**
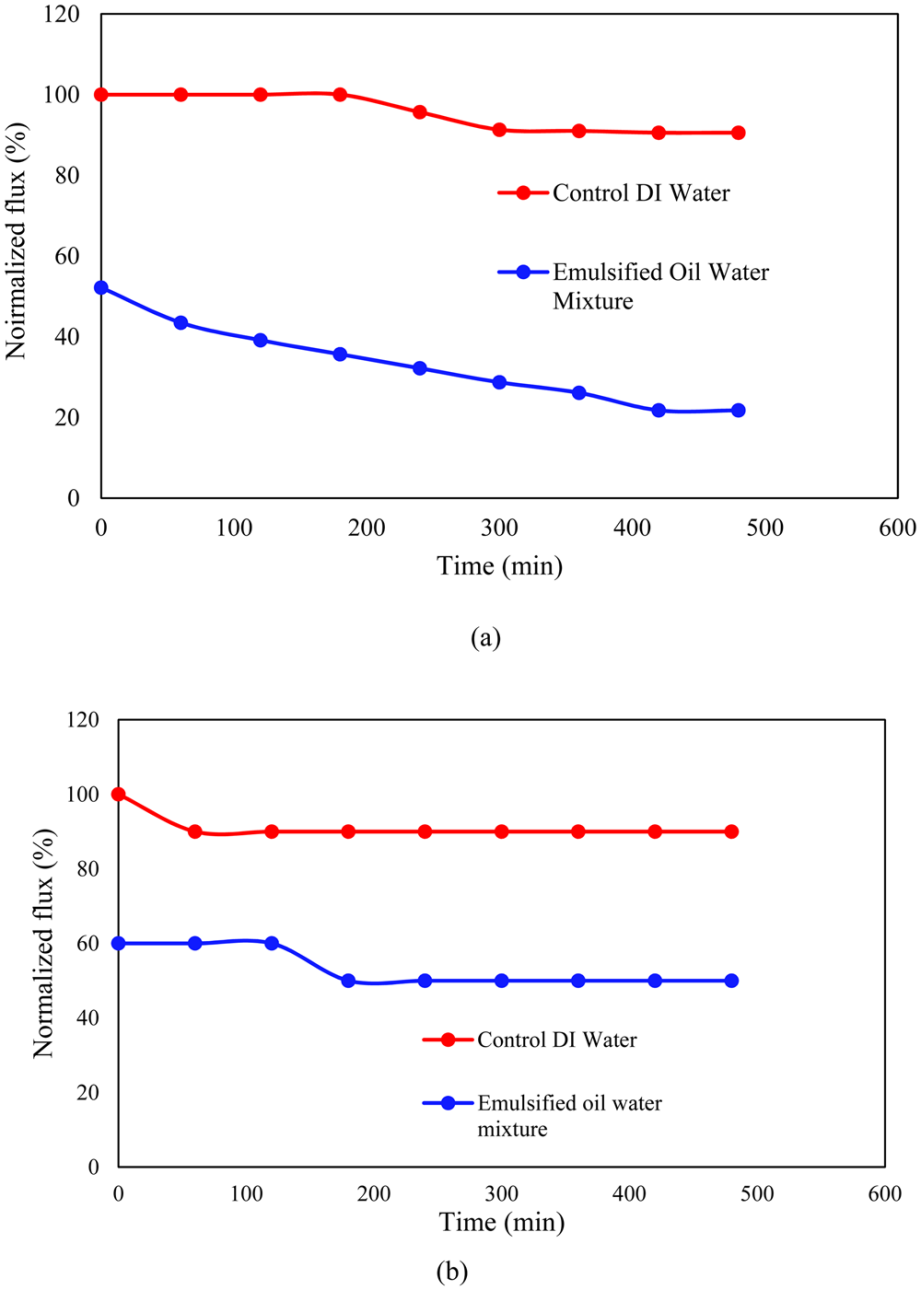
Comparison of the fouling behaviors of emulsified oil particles in the FO operations using (**a**) PES-TFC and (**b**) PES-TFC-g-PMAPS membranes under the AL-DS mode. Feed: DI water or 10,000 ppm oil water solution. Draw: 2 M NaCl solution.

The benefits of coating a zwitterion copolymer layer onto the bottom of the TFC FO membrane were demonstrated by comparing the fouling behavior between the single-skinned and double-skinned membrane under the AL-DS operation mode. Both membranes were operated at a similar initial water flux of approximately 14 LMH. As shown in Figure 9, the single-skinned membrane has a faster decline in water flux over time compared with that of double-skinned membrane when treating an emulsified oil-water mixture of 10,000 ppm. The presence of zwitterion copolymer layer shows protection over the support layer with lower fouling propensity. The emulsified oil particles tend to foul faster on the TFC surface if there is no zwitterion copolymer layer. The presence of the zwitterion copolymer layer reduces the amount of emulsified oil particles approaching the TFC layer so that the newly developed double-skinned membrane can overcome the fast fouling problem of TFC membranes in oil-water separation.

**Figure 10:**
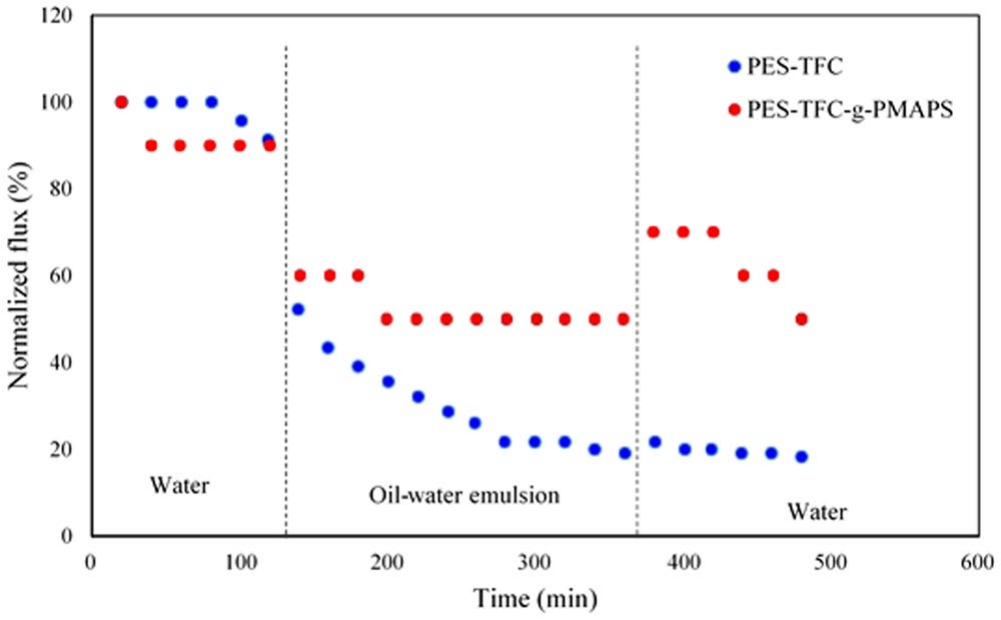
Comparison between the water flux recovery of single-skinned and double-skinned TFC membranes during three steps: (i) pure water flux for 120 min, (ii) oil-water emulsion water flux for 260 min and (iii) pure water for 120 min after washing with DI water.

In addition to the superior antifouling and ICP properties, the PES-TFC-g-PMAPS membrane also shows its advantage over the PES-TFC membrane in prolonged experimental process. Figure 10 compares the water flux profile of two membranes over a period of 480 min in three-step filtration process using DI water and oil-water solution. The significant flux decrease in the PES-TFC membrane is resulted from internal fouling due to the entering of oil particles from the feed into the porous support and external fouling due to the deposition of oil particles on the membrane surface. As a result, both ICP effect and transport resistance increase. Comparing with the PES-TFC membrane, the PES-TFC-g-PMAPS membrane exhibited lower degree of flux decline for the same filtration period. After being rinsed with DI water for 120 min, the water fluxes of PES-TFC and PES-TFC-g-PMAPS membranes were recovered to 21% and 70%, respectively. At the end of 480-min operation time, both water fluxes of PES-TFC and PES-TFC-g-PMAPS membranes were further decreased to 18% and 50% (compared to their respective initial flux), respectively. The higher water flux recovery of PES-TFC-g-PMAPS membrane can be explained by the superior hydrophilic properties of PMAPS which plays as an antifouling layer to prevent internal fouling and reduce the effects of ICP. When PMAPS is immersed into water, the PMAPS chain is hydrated and water molecules are thus trapped within the PMAPS and forms a water layer. This water layer could prevent oil from adsorbing onto the membrane surface^33^.

## Conclusion

A high quality water with oil rejection >99.9% can be obtained using the double-skinned membrane at a reasonably good water flux of 13.7 LMH using 2 M NaCl as the draw solution. The depositions of PA and PMAPS layers onto the PES support were further confirmed by the analyses of chemical changes by FESEM, AFM, FTIR and EDX. After filtration for 480 min for both DI and oil-water emulsion, the water fluxes of both single- and double-skinned TFC membranes decreased to 18% and 50% of original flux, respectively. The higher water flux recovery of double-skinned membrane can be explained by the superior hydrophilic properties of PMAPS which plays as an antifouling layer to prevent internal fouling and reduce the effects of ICP. This experimental work can provide a versatile approach for the design and fabrication of antifouling double-skinned TFC membrane in molecular level for oily wastewater treatment.

## Electronic supplementary material

Supplementary information
