# Supplementary material for: Anti-Fouling Double-Skinned Forward Osmosis Membrane with Zwitterionic Brush for Oily Wastewater Treatment
Source: Sci Rep. 2017 Jul 31;7:6904. doi: 10.1038/s41598-017-07369-4 (PMC5537261; doi:10.1038/s41598-017-07369-4)
Supplement: Supplementary file 1 — Supplementary information [file 41598_2017_7369_MOESM1_ESM.doc]

**Anti-Fouling Double-skinned Forward Osmosis Membrane with Zwitterionic Brush for Oily Wastewater Treatment**

Chi Siang Ong^a*^, Bader Al-anzi^a^, Woei Jye Lau^b^, Pei Sean Goh^b*^, Gwo Sung Lai^b^, Ahmad F. Ismail^b^, Yue Seong Ong^c^

^a^Department of Environment Technology and Management, College of Life Sciences, Kuwait University, Kuwait

^b^Advanced Membrane Technology Research Centre (AMTEC), Universiti Teknologi Malaysia, 81310 Skudai, Johor, Malaysia.

^c^Faculty of Engineering and the Built Environment, SEGi University, 47810 Petaling Jaya, Selangor, Malaysia

*Corresponding authors: Email address: ongchisiang@gmail.com (C.S. Ong), peisean@petroleum.utm.my (P.S. Goh)

**Supplementary information**

**Preparation of dope solution**

The dope solution used for membrane substrate fabrication contained 18 wt.% PES, 1 wt.% PVP and 81 wt.% NMP. To make the dope solution, 1.0 wt.% PVP was first added into NMP solvent. The mixture was then mechanically stirred at 400 rpm and 60^o^C until a homogenous mixture was formed. PES pellets was then added to the mixture and continuously stirred for 3 h until a homogenous solution was formed. The homogenous dope solution was left at 25°C for 24 h to eliminate air bubbles trapped within the dope solution.

**Synthesis of zwitterionic polymer**

The zwitterionic monomers, MAPs were synthesized according to the method reported in literature . 3.774 g (equivalent to 0.024 mol) of DMAEMA was firstly dissolved in 13 mL of acetone and placed into a 250 mL flask on a magnetic stirrer. 2.442 g (0.02 mol) of 1,3-propanesultone was added drop wise to the mixture in the flask under N_2_ atmosphere at room temperature. The reaction was conducted at room temperature for 4 h. A crude white powder product was produced at the end of the reaction. It was then washed with ethanol followed by precipitation in diethyl ether to remove the unreacted reagents.

**Fabrication of membrane substrate**

The membrane substrate was cast manually on a glass plate with masking tape placed on both sides of the plate to obtain a desired thickness of 200 ± 20 µm. A glass rod was used to pull the dope solution across the glass plate followed by immersion into a RO water coagulation bath for phase inversion to take place. Once the polymeric film was peeled off from the glass plate, it was transferred to another RO water bath and further immersed for at least 24 h to remove residual solvent before it was used for TFC membrane making.

**Fabrication of double-skinned forward osmosis membrane**

**Figure 1** (in the main text) shows the fabrication process of the double-skinned FO membrane. Firstly, the synthesized zwitterion polymer was coated at the bottom surface of PES support membrane. 1 mL of 2-bromoisobutyrate bromide in 15 mL of diethyl ether was added into 0.75 mL of pyridine in 25 mL of diethyl ether (referred as solution A). The solution was stirred at iced water bath (0^o^C) for 1 h and ultrasonicated for 30 min. Secondly, 1 g of MAPS, 5 mL of ethanol, 0.69 g of HMImCl, 1.39 mg of CuBr, 2.76 mg of Bpy, and 1.36 µL of EBiB were mixed together and ultrasonicated for another 30 min (referred as solution B). Prior to the surface grafting, 50 mL of 0.1 wt.% of glycerol water solution was allowed to come into contact with the bottom surface for 3 h to achieve surface hydroxylation. The excess solution was drained off and the remaining droplets were removed by tissue papers. The surface grafting was accomplished by pouring solution A on the membrane surface (Area: 0.01 cm^2^) for 30 min followed by solution B for 10 min. It was then placed at 0 ^o^C for 1 h followed by 60 ^o^C for 30 min. The resulting membrane was stored in RO water for 24 h to remove the excessive residue and ensure the PMAPS was firmly formed on the membrane surface.

In the second stage, the polyamide (PA) layer was deposited onto the top of the PMAPS grafted PES support via interfacial polymerization process between MPD and TMC, according to method as reported in literature [^3^](#_ENREF_3). The PMAPS grafted PES support was placed on a glass plate and a rubber roller was carefully applied to remove water droplets on the top of the membrane surface. The presence of water droplets may interfere with the formation of PA layer. A rubber frame was placed on top of the membrane substrate followed by a glass frame and clamped together using four paper clips. The process has to be carried out carefully to prevent any damage to the membrane substrate. At first, 40 mL of 2 w/v% MPD aqueous solution was poured onto the top of PES substrate and the solution was held on the surface for 10 min to ensure the penetration of MPD monomers to the pores of substrate. After that, the aqueous solution was drained off and its residual droplets on the surface were removed using rubber roller. 40 mL of 0.1 w/v% TMC in cyclohexane solution was then poured on the top surface of the substrate and allowed to have a reaction time of 1 min to ensure the formation of the PA layer. The organic solution was drained off and the substrate was dried in ambient condition to evaporate excess cyclohexane. Similar procedure was used for fabrication of single skinned TFC membrane. At the end of the process, the resulting membrane (PES-TFC-g-PMAPS) was further post-treated in an oven at 60 ^o^C for 8 min. Finally, the membrane was left at room temperature for 30 min before storing in a RO water container until further use.

**Preparation of oil emulsion feed solution and its calibration graph**


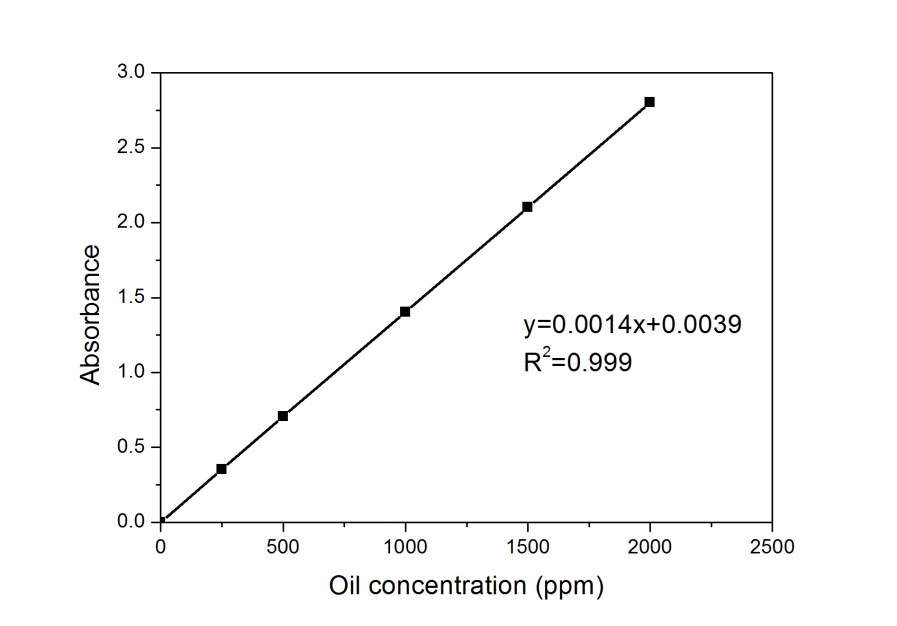
The emulsion was prepared by mixing Red Eagle commercial oil and sodium dodecylbenzenesulfonate (SDS) at the ratio of 9:1. The solution was then blended by a speed blender (Model: BL 310AW, Khind) for 2 min with an agitation speed of 50 Hz at room temperature. The relation between absorbance and oil concentration is found to be linear (using UV-vis spectrophotometer) as shown in **Figure S1**. The same relation has been used for measuring unknown oil concentration in each permeate.

**Figure S1**. Calibration graph between absorbance and oil concentration

| **Table S1:** Performance Comparison between Single- and Double-Skinned TFC Membranes | | | | | |
| --- | --- | --- | --- | --- | --- |
| **Flat Substrate** | **Top layer** | **Bottom layer** | **Testing conditions** | **Performance** | **References** |
| **sPPSU/PEG400/NMP** | 2.0 wt% MPD solution; 0.05 wt% TMC in hexane | 2.0 wt% MPD solution; 0.05 wt% TMC in hexane | FS: 100 ppm boron solution  DS: 1000 ppm NaCl draw solution | Salt rejection: ~95% (both TFC at top or bottom surface)  Boron rejection: 41.7% (TFC layer at top surface) and 43.1% (TFC layer at top and bottom surface)  Double-skinned TFC membrane achieved higher boron rejection but lower water flux compared to single-skinned TFC membrane in both operation mode (FO and PRO) | Luo et al.[^4^](#_ENREF_4) |
| **PSF-DMF (embedded with PET mesh)** | 2 g/L dopamine solution  (contact time: 0-6 h)  4 wt% MPD and 0.1 wt% TMC | 2 g/L dopamine solution  (contact time: 0-6 h)  4 wt% MPD and 0.1 wt% TMC | DS: 1 M NaCl  FS: 200 ppm sodium alginate (SA) | Double-skinned TFC membrane coated with dopamine solution showed higher water flux (1.94 LMHbar^-1^) than single-skinned TFC membrane (1.76 LMHbar^-1^) when contact time was set at 2 h. Further increase in contact time to 6 h however slightly reduced water flux to 1.93 LMHbar^-1^.  Salt rejection remained relatively stable at ~97% as the contact time of dopamine solution increased from zero to 6 h. | Liu et al. [^5^](#_ENREF_5) |
| **PSf/NMP/PVP/LiCl** | 2 g/L Dopamine hydrochloride (DA, 98%), 0.01-0.05 wt/v% carbon nanotubes (CNTs) and 0.1 wt/v% trimesoyl chloride  (TMC, 98%) in n-hexane | 2 g/L Dopamine hydrochloride (DA, 98%), 0.01-0.05wt/v% carbon nanotubes (CNTs) and 0.1 wt/v% trimesoyl chloride (TMC, 98%) in n-hexane | FS: DI water  DS: 2 mol/L MgCl_2_  5mg/L humic acid (HA) | Water flux increased from 6.5 to 8 LMH when CNTs loading increased from 0.01 wt/v% to 0.05 wt/v% but reduced to 7 LMH when CNTs loading further increased to 0.1 wt/v% compared to pristine TFC (5.8 LMH) under FO mode.  Water flux increased from 8 to 12 LMH when CNTs loading increased from 0.01 wt/v% to 0.05 wt/v% but reduced to 10 LMH when CNTs loading further increased to 0.1 wt/v% as compared to pristine TFC (8 LMH) under PRO mode.  TFN with CNTs loading of 0.05 wt/v% achieved higher flux recovery of 93.5% than pristine TFC double-skinned membrane of 84.8% using 5 mg/L humic acid solution as foulant. | Song et al.[^6^](#_ENREF_6) |
| **Cellulose Acetate (CA)/NMP**  **Polysulfone (PSf)/NMP** | 2.0 wt% MPD solution; 0.1 wt% TMC in hexane; Sulfonated poly(phenylene oxide) (SPPO) | 2.0 wt% MPD solution; 0.1 wt% TMC in hexane; Sulfonated poly(phenylene oxide) (SPPO) | FS: Seawater  DS: 2M NaCl | Double-skinned CA membrane exhibited lower PWP and salt permeability of 0.59 LMHbar^-1^ and 0.19 LMHbar^-1^, respectively compared to single-skinned CA membrane of 1.18 LMHbar^-1^ and 0.41 LMHbar^-1^, respectively.  Double-skinned PSf membrane exhibited lower PWP and salt permeability of 0.59 LMHbar^-1^ and 0.06 LMHbar^-1^, respectively as compared to single-skinned PSf membrane of 1.21 LMHbar^-1^ and 0.13 LMHbar^-1^, respectively. | Zhou & Lee et al.[^7^](#_ENREF_7) |
| **PAN, LiCl, ethanol and DMF** | 1.5% (w/v) MPD; 0.05% (w/v) TMC | Nexar® co-polymer | FS: Oil-water emulsion (200,000 ppm)  DS: 2000 ppm NaCl | PWP of the double-skinned membrane (1.29 LMH/bar) was slightly lower than that of the single-skinned membrane (1.37 LMH/bar) under the RO tests.  Salt rejection of the double-skinned membrane remained almost the same with the single-skinned membrane at approximate 88%.  Double-skinned membrane has high oil rejection of >99.9%.  The resultant double-skinned membrane exhibits a high water flux of 17.2 LMH and a low reverse salt transport of 4.85 gMH under PRO mode using 0.5 M NaCl as the draw solution and DI water as the feed. | Duong et al.[^8^](#_ENREF_8) |
| **18 wt.% PAN and 2 wt.% LiCl in DMF** | Poly(allylamine hydrochloride) (PAH) / poly(sodium 4-styrenesulfonate (PSS)/ glutaraldehyde  (GA)  Cross-linked Layer-by-Layer (xLbL) coating | Poly(allylamine hydrochloride) (PAH) / poly(sodium 4-styrenesulfonate (PSS)/ glutaraldehyde  (GA)  Cross-linked Layer-by-Layer (xLbL) coating | FS: MgCl_2_, DI water  DS: MgCl_2_, NaCl | Both the top and bottom skins contributed to the overall water resistance of the double-skinned xLbL membranes. However, the overall salt rejection was mainly determined by the top skin.  The double-skinned xLbL FO membranes achieved excellent FO water flux and low specific reverse solute diffusion.  The double-skinned xLbL membranes demonstrated much better antifouling performance compared to the single-skinned counterpart. | Qi et al.[^9^](#_ENREF_9) |

*FS: Feed solution; DS: Draw solution

**References**
